# Allele-Selective Genome Editing of the Human Leukocyte Antigen Locus in Human Dental Pulp Cells Using Zinc Finger Nucleases

**DOI:** 10.7759/cureus.84920

**Published:** 2025-05-27

**Authors:** Izumi Kuroda, Tomoko Kawaguchi, Shunji Chikusa, Shota Ishii, Hitomi Aoki, Tsutomu Motohashi, Takahiro Kunisada, Masatake Osawa, Ken-ichi Tezuka

**Affiliations:** 1 Anesthesiology, Laboratory of Molecular Design and Synthesis, Gifu University Graduate School of Medicine, Gifu, JPN; 2 Stem Cell and Regenerative Medicine, Gifu University Graduate School of Medicine, Gifu, JPN; 3 Oral and Maxillofacial Surgery, Gifu University Graduate School of Medicine, Gifu, JPN; 4 Stem Cell Biology, Laboratory of Molecular Design and Synthesis, Gifu University Graduate School of Medicine, Gifu, JPN; 5 Dermatology, Laboratory of Molecular Design and Synthesis, Gifu University Graduate School of Medicine, Gifu, JPN; 6 Dentistry, Center for One Medicine Innovative Translational (COMIT) Research, Gifu University Institute for Advanced Study, Gifu, JPN

**Keywords:** dental pulp cell, engraftment, genome editing, human leukocyte antigen (hla), whole genome sequencing (wgs), zinc finger nucleases

## Abstract

Human dental pulp cells (DPCs) hold promise for cell-based therapies, but their allogeneic use is limited by human leukocyte antigen (HLA) incompatibility. Utilizing HLA haplotype-homozygous (HHH) donors can improve compatibility, yet sourcing donors for diverse haplotypes remains challenging. Generating pseudo-HHH cells by disrupting a specific HLA allele via gene editing offers a potential solution. This study aimed to establish zinc finger nuclease (ZFN)-mediated gene editing for allele-specific disruption of HLA-A in DPCs. We designed ZFNs to target the HLA-A*02:01 allele in DP144 DPCs (HLA-A*02:01/A*33:03, homozygous at HLA-B, -C, -DR). Following ZFN transfection and fluorescence-activated cell sorting (FACS)-mediated cell enrichment, allele-specific modifications were assessed using targeted deep sequencing, whole-genome sequencing (WGS) for off-target analysis including structural variants (SVs), and next-generation sequencing (NGS)-based HLA typing. Results demonstrated efficient (>96% indels) and specific disruption of the targeted HLA-A*02:01 allele, with minimal modification of the HLA-A*33:03 allele. Whole-genome sequencing revealed overall genomic stability, although two minor deletion-type SVs were detected in non-coding regions. Importantly, NGS HLA typing confirmed the integrity of the non-targeted HLA-A allele and other key HLA loci. These findings demonstrate the feasibility of using ZFNs to generate pseudo-HHH DPCs by allele-specific HLA knockout, providing a potential strategy to expand the donor pool for DPC-based therapies. However, careful assessment of genomic integrity is crucial for future clinical translation.

## Introduction

Human dental pulp cells (DPCs) are readily accessible mesenchymal cell-like populations isolated from extracted wisdom teeth or exfoliated deciduous teeth [1]. Characterized by their ease of propagation in extended culture and their capacity for induced differentiation into diverse lineages, DPCs represent an attractive source for cell-based regenerative medicine applications [2]. Furthermore, the ethical neutrality and availability of DPCs as clinical waste have spurred national initiatives for DPC-derived induced pluripotent stem cell (iPSC) banking, underscoring their practical utility [3]. 

A major challenge in cell-based therapies is overcoming host immunological barriers, primarily mediated by human leukocyte antigen (HLA) mismatches. The HLA proteins are categorized into classes I, II, and III; aligning class I (specifically HLA-A and -B) and class II (HLA-DR) is critical for mitigating graft rejection [4]. A realistic approach to enhance compatibility involves using cells from HLA haplotype-homozygous (HHH) donors, where the HLA-A, -B, and -DR loci are genetically homozygous. These cells can be transplanted into recipients sharing at least one identical haplotype [5]. To advance the allogeneic application of DPCs, our group previously established HHH DPC lines covering approximately 20% of the Japanese population [6]. However, expanding this coverage is challenging due to the difficulty in recruiting HHH donors with rare HLA haplotypes, unlike iPSC banking efforts, which have achieved broader coverage for the most frequent haplotypes [7]. Identifying donors for rarer haplotypes requires screening vast numbers of individuals. 

To address the limitation of HHH donor availability, generating 'pseudo-HHH' DPCs by disrupting a specific HLA allele via gene editing presents a potential solution [8]. Genome editing technologies such as zinc finger nucleases (ZFNs), transcription activator-like effector nucleases (TALENs), and clustered regularly interspaced short palindromic repeats (CRISPR)/Cas9 allow precise DNA modification and have been applied in various cell types; however, their use in DPCs is less explored. Previous studies have shown the feasibility of gene manipulation in DPCs using ZFNs for targeted insertion or CRISPR/Cas9 for enhancing regeneration or knocking out specific genes [9,10]. However, HLA gene editing in DPCs specifically for allogeneic transplantation remains largely uninvestigated. 

This study aimed to establish a methodology for generating pseudo-HHH DPCs by selectively ablating a specific HLA-A allele using ZFNs. The ZFNs were chosen over CRISPR/Cas9 due to their potential for greater specificity when targeting highly homologous sequences, such as HLA alleles, where designing unique guide RNAs for CRISPR/Cas9 can be challenging [8]. The ZFNs utilize customizable zinc finger domains for target recognition, enabling more precise targeting of non-homologous sequences necessary for knocking out a single HLA allele while preserving the other. We specifically targeted the HLA-A*02:01 allele in the DP144 DPC line (heterozygous at HLA-A but homozygous at HLA-B, -C, and -DR) [6] to create pseudo-HHH cells. The rationale for this strategy was to generate HHH-like cells by ablating the high-frequency HLA-A*02:01 allele while preserving the less common HLA-A*33:03 allele through genome editing. This approach aims to expand the potential donor pool, particularly for recipients carrying less frequent HLA alleles like A*33:03, by increasing the availability of suitably matched cells. Our findings demonstrate the feasibility of this ZFN-based, allele-specific ablation in DPCs, offering a potential strategy to enhance the utility of partially matched donors and providing insights toward developing broadly immune-compatible cell therapies

This article was previously presented as a poster at the 21st Congress of The Japanese Society for Regenerative Medicine on March 19, 2022, at The 40th Annual Meeting of The Japanese Society for Bone and Mineral Research on July 22, 2022, and at the 23rd Congress of The Japanese Society for Regenerative Medicine on March 23, 2024.

## Materials and methods

Study design and ethical compliance

This study was designed to investigate the feasibility of allele-specific genome editing at the HLA-A locus in DPCs using ZFNs. The objective was to selectively ablate the HLA-A*02:01 allele to create pseudo-HHH cells while preserving expression of the HLA-A*33:03 allele. Ethical approval for the use of human-derived dental pulp tissues was obtained from the Gifu University Hospital Medical Research Ethics Review Committee (approval no. 29-323). Written informed consent was obtained from a donor prior to tooth extraction. We obtained permission to use these cells for whole-genome analyses (certification no. 2023-322).

Isolation, culture, and HLA typing of dental pulp cells

The DPCs were isolated from the pulp of surgically extracted third molars and expanded in culture. The DP144 cell line [6], characterized as HLA-A*02:01:01/33:03:01, B*44:03:01:10, C*14:03:01:01, DRB1*13:02:01, DRB3*03:01:01, DQA1*01:02:01, DQB1*06:04:01, DPA1*02:02:02, and DPB1*05:01, was used for all experiments. High-resolution HLA typing was performed by GenoDive Pharma Inc. (Atsugi, Japan), using the AllType next-generation sequencing (NGS) 11-Loci Amplification Kit (One Lambda, Los Angeles, CA, USA), followed by sequencing on the Ion S5 platform. Cells were maintained in MSCGM™ Mesenchymal Stem Cell Growth Medium (Lonza, Basel, Switzerland; #PT-3001) at 37 °C in a humidified incubator with 5% CO₂ and 95% air. Subculturing was performed at 70% to 80% confluence using TrypLE Select (Thermo Fisher Scientific, MA, USA; #12605028).

ZFN design, plasmid preparation, and electroporation

Custom ZFNs specifically targeting exon 3 of the HLA-A*02:01 allele were designed and synthesized by Sigma-Aldrich (CompoZr Custom ZFN, Merck KGaA, St. Louis, MO, USA), in collaboration with Sangamo Biosciences Inc. (Richmond, CA, USA), which performed MEL1 reporter yeast assays to assess cleavage activity and specificity (Table 1). The selected ZFN pair (HLAA-491a1/HLAA-r484a1) was chosen based on high target-specific activity and minimal homology to the HLA-A*33:03 allele. The ZFN-encoding plasmids were amplified in Escherichia coli and purified using the Qiagen Plasmid Maxi Kit (Qiagen GmbH, Hilden, DEU). For transfection, DP144 cells were harvested at 70% to 80% confluence, resuspended in OptiMEM (Thermo Fisher Scientific), and mixed with 5 µg of ZFN plasmid DNA. Electroporation was conducted using a NEPA21 type II electroporator (NEPA GENE, Ichikawa, Chiba, Japan) with the following optimized parameters: 150 V pulse voltage, 5 ms pulse width, 50 ms inter-pulse interval, and 40% decay rate. Cells were immediately transferred into MSCGM medium and incubated under standard culture conditions for recovery.

ZFN-mediated disruption of HLA-A*02 expression and enrichment of edited cells

To identify and isolate cells with disrupted HLA-A02 expression, transfected cells were subjected to fluorescent-activated cell sorting (FACS). For sorting, a double-staining protocol was employed using phycoerythrin (PE-conjugated) anti-HLA-A02 antibody (MBL Life Science Inc., Tokyo, Japan; #ab79523), rabbit anti-pan-HLA-A monoclonal antibody (Abcam Ltd., Cambridge, GBR; catalog #ab52922), and Alexa Fluor 488-conjugated goat anti-rabbit IgG (Thermo Fisher Scientific). Cells were resuspended in a staining buffer containing 10% fetal calf serum (FCS), 0.01% sodium azide (NaN₃), and 1 mM ethylenediaminetetraacetic acid (EDTA) in phosphate-buffered saline (PBS) and incubated with antibodies for 30 min on ice. After two rounds of cell sorting on a BD FACS Aria system (Becton, Dickinson and Co., Franklin Lakes, NJ, USA), HLA-A02-negative and pan-HLA-A-positive cells were isolated with >99% purity. Unstained cells and single-stain controls were used to establish sorting gates. Sorted cells were collected in polypropylene tubes containing MSCGM medium and expanded for downstream analysis.

Targeted amplicon deep sequencing

Genomic DNA was extracted using the NucleoSpin® DNA RapidLyse kit (MACHEREY-NAGEL, Düren, DEU) to determine the mutational profile at the ZFN-targeted HLA-A loci. Nested PCR was performed using KOD One Plus (Toyobo Co., Ltd., Osaka, JPN) and HiDi DNA polymerase (myPOLS Biotec GmbH, Konstanz, DEU) to amplify a ~200 bp region encompassing the ZFN cleavage site. Adapter and index sequences were added using a MiSeq v3 reagent kit (Illumina, San Diego, CA, USA), and sequencing was carried out on the Illumina MiSeqDx (Illumina Inc., San Diego, CA, USA) platform. The specific primer sequences used are provided upon request. The PCR products were purified using the QIAprep Spin Gel Purification Kit (Qiagen GmbH). Sequencing data were analyzed using Cas-Analyzer [11] and the Sequence Interrogation and Quantification (SIQ) software [12] to quantify insertion/deletion (indel) events and mutation frequencies at the edited sites.

Whole-genome sequencing (WGS) and read processing

To evaluate global genomic integrity, WGS was performed on DNA extracted from both ZFN-edited and control DP144 cells. The PCR-free libraries were prepared using the TruSeq DNA PCR-Free Library Prep Kit (Illumina Inc.) and sequenced using a paired-end 150 bp strategy on the Illumina NovaSeq 6000 platform. Quality assessment was conducted with FastQC (v0.11.7; Babraham Bioinformatics, Cambridge, GBR) [13], and trimming of low-quality bases (Phred score <20) and adapter sequences was performed using Trimmomatic (v0.38; Usadel Lab, Aachen, DEU) [14]. Reads were aligned to the GRCh38 reference genome and converted to a sorted binary alignment map (BAM) format using Picard SortSam and MarkDuplicates tools (https://broadinstitute.github.io/picard/).

Copy number variation (CNV) analysis

Somatic copy number changes were evaluated using CNVkit (v0.7.4) [15] in whole-genome mode. Fixed 10-kb genomic bins were used to compute read depth across the genome. A control reference was built using the unedited DP144 sample, and the edited sample was normalized and segmented relative to this reference. The CNV plots were generated using the scatter and diagram functions in the CNVkit. Circos plots (generated with Circa v2.0; OMGenomics Labs, San Francisco, CA, USA) were used to visualize CNV differences across all chromosomes.

Somatic structural variant (SV) calling

To detect gene-editing-induced SVs, somatic SV calling was performed using four independent algorithms: Manta (v1.6.0) [16], Delly (v1.33) [17], GRIDSS2 (v2.13.2) [18], and SvABA (v1.1.4) [19]. Gene-edited samples were designated as the tumor, and the parental DP144 cells served as matched normal. Variants were filtered by quality (PASS status or QUAL >30), supporting reads, and variant type. High-confidence SVs detected by at least two callers were merged using SURVIVOR (v1.0.7) [20] with a maximum breakpoint distance of 1,000 bp and matching variant types. Allele frequencies (AF) and read depths (DP) were annotated from BAM files. SV visualization was performed using Samplot [21] and Circa (OMGenomics Labs).

Off-target site prediction

In silico prediction of potential off-target cleavage sites for the HLAA-491a1/HLAA-r484a1 ZFN pair was performed using CasOFFinder [22] and PROGNOS [23] tools, allowing up to five mismatches. Candidate loci were subsequently analyzed using targeted sequencing to assess indel frequency and validate specificity.

HLA re-typing and functional validation

To verify pseudo-HLA haplotype homozygosity, long-range PCR (>5 kbp) was used to amplify the full coding sequences of 11 HLA loci using the AllType NGS 11-Loci Amplification Kit (One Lambda, CA, USA). Libraries were sequenced using the Ion S5 platform (Thermo Fisher Scientific). The SIQ tool was used to evaluate sequence integrity, and mutational profiles were visualized with SIQPlotteR tornado plots [12]. The HLA class II loci were specifically assessed for unintended alterations.

## Results

Selection and validation of ZFNs for allele-specific targeting

**Figure 1:**
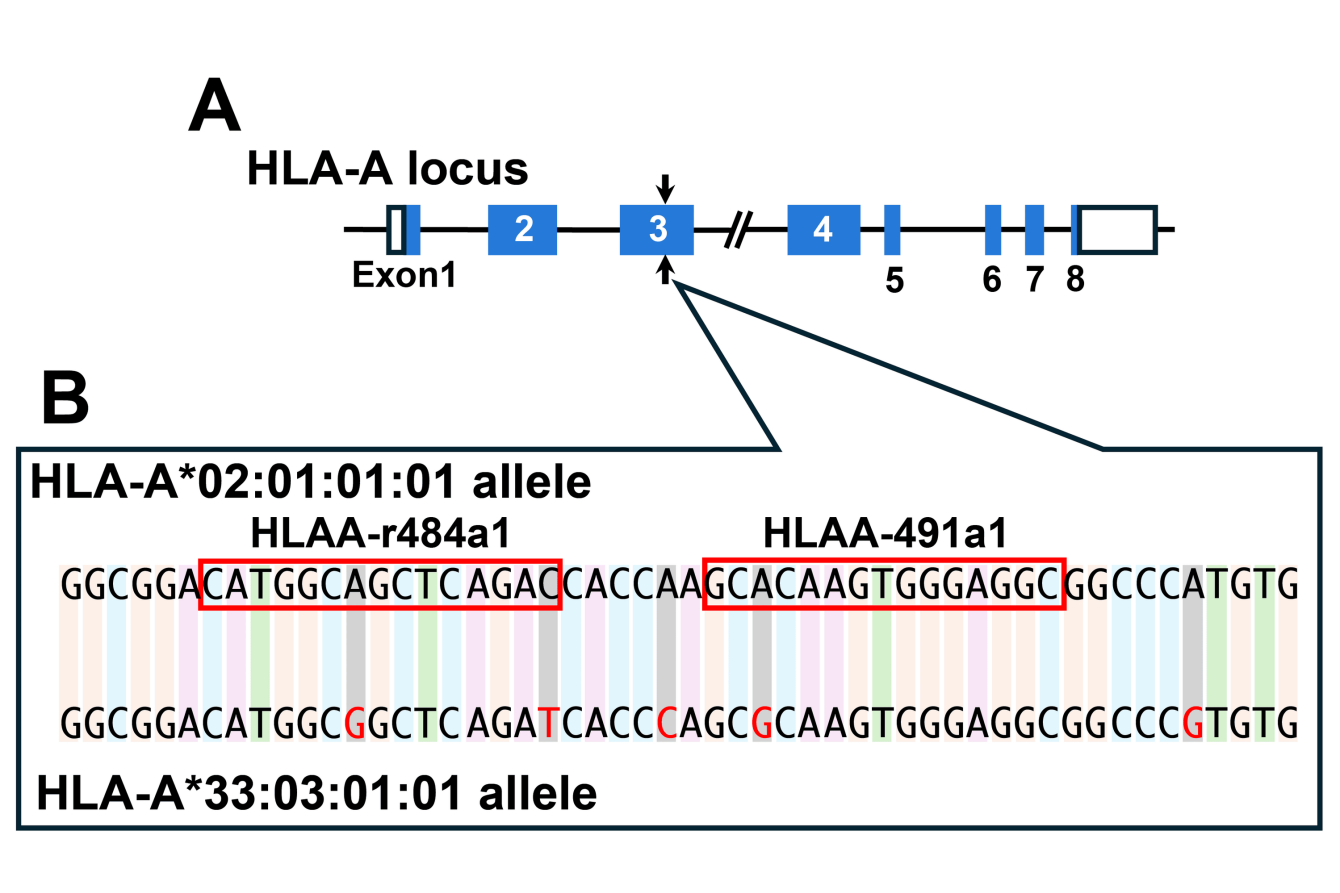
Schematic representation of the ZFN target site within the HLA-A*02 allele A: Diagram of the HLA-A genomic locus showing exon structures (blue boxes) and untranslated regions (white boxes). The ZFN cleavage site within exon 3 is indicated by vertical arrows. B: Nucleotide sequence comparison between HLA-A*02:01:01:01 (top) and HLA-A*33:03:01:01 (bottom) alleles at the ZFN target region. Red boxes highlight the binding sites of the HLAA-r484a1 and HLAA-491a1 ZFN pair. Nucleotides in HLA-A*33:03:01:01 that differ from HLA-A*02:01:01:01 are shown in red. ZFN: Zinc finger nuclease, HLA: Human leukocyte antigen

To minimize off-target cleavage events, we employed high-fidelity obligate heterodimeric FokI domains in our ZFN design. These domains have been demonstrated to reduce off-target effects by preventing DNA digestion mediated by homodimerization [24]. To target the HLA-A*02:01 allele specifically while minimizing potential cleavage of the homologous HLA-A*33:03 allele, 46 candidate ZFN pairs targeting exons 2 and 3 were designed. Selection prioritized pairs with maximal mismatches against the HLA-A*33:03 sequence. The ZFN pair HLAA-491a1/HLAA-r484a1, targeting exon 3 (Figure 1A) and exhibiting high MEL I activity and three nucleotide mismatches compared to HLA-A*33:03 (Figure 1B; Table 1), was selected for subsequent experiments. 

**Table 1:** List of candidate ZFN pairs and their MEL I assay results *ND: Nuclease activity was not detectable ZFN: Zinc finger nuclease

Right ZFN	Target sequence	Left ZFN	Target sequence	Number of mismatches in HLA-A*33	MEL I assay*
HLAA-225a1	GTGGACCTGGGGACC	HLAA-r219a1	GAGTCTGTGAGTGGG	1	ND
HLAA-228a1	GACCTGGGGACCcTGCGCG	HLAA-r222a1	GGTGAGTCTGTGAGTGGG	1	ND
HLAA-230a1	CCTGGGGACCCTGcGCGGCT	HLAA-r224a1	TCGGTGAGTCTGTGAGTG	1	ND
HLAA-234a1	GGGACCCTGCGCGGCT	HLAA-r227a1	CACTCGGTGAGTCTG	1	33.50%
HLAA-491a1	GCACAAGTGGGAGGC	HLAA-r484a1	GTCTGAGCTGCCATG	3	76.20%
HLAA-494a1	CAAGTGGGAGGC	HLAA-r488a1	GGTGGTCTGAGCTGCCATG	2	64.80%
HLAA-496a1	AGTGGGAGGCGGCCCATG	HLAA-r490a1	TTGGTGGTCTGAGCT	4	ND
HLAA-496a1	AGTGGGAGGCGGCCCATG	HLAA-r489a1	TGGTGGTCTGAG	3	ND
HLAA-498a1	TGGGAGGCGGCCCATGTG	HLAA-r492a1	GCTTGGTGGTCTGAG	3	ND
HLAA-501a1	GAGGCGGCCCATGTG	HLAA-r495a1	TGTGCTTGGTGGTCTGAG	4	ND
HLAA-504a1	GCGGCCCATGTGGCGGAG	HLAA-r497a1	CTTGTGCTTGGTG	3	ND
HLAA-528a1	AGAGCCTACCTGGAGGGC	HLAA-r522a1	GCTCCGCCACATGGGC	1	ND

ZFN-mediated disruption of HLA-A*02 expression and enrichment of edited cells

**Figure 2:**
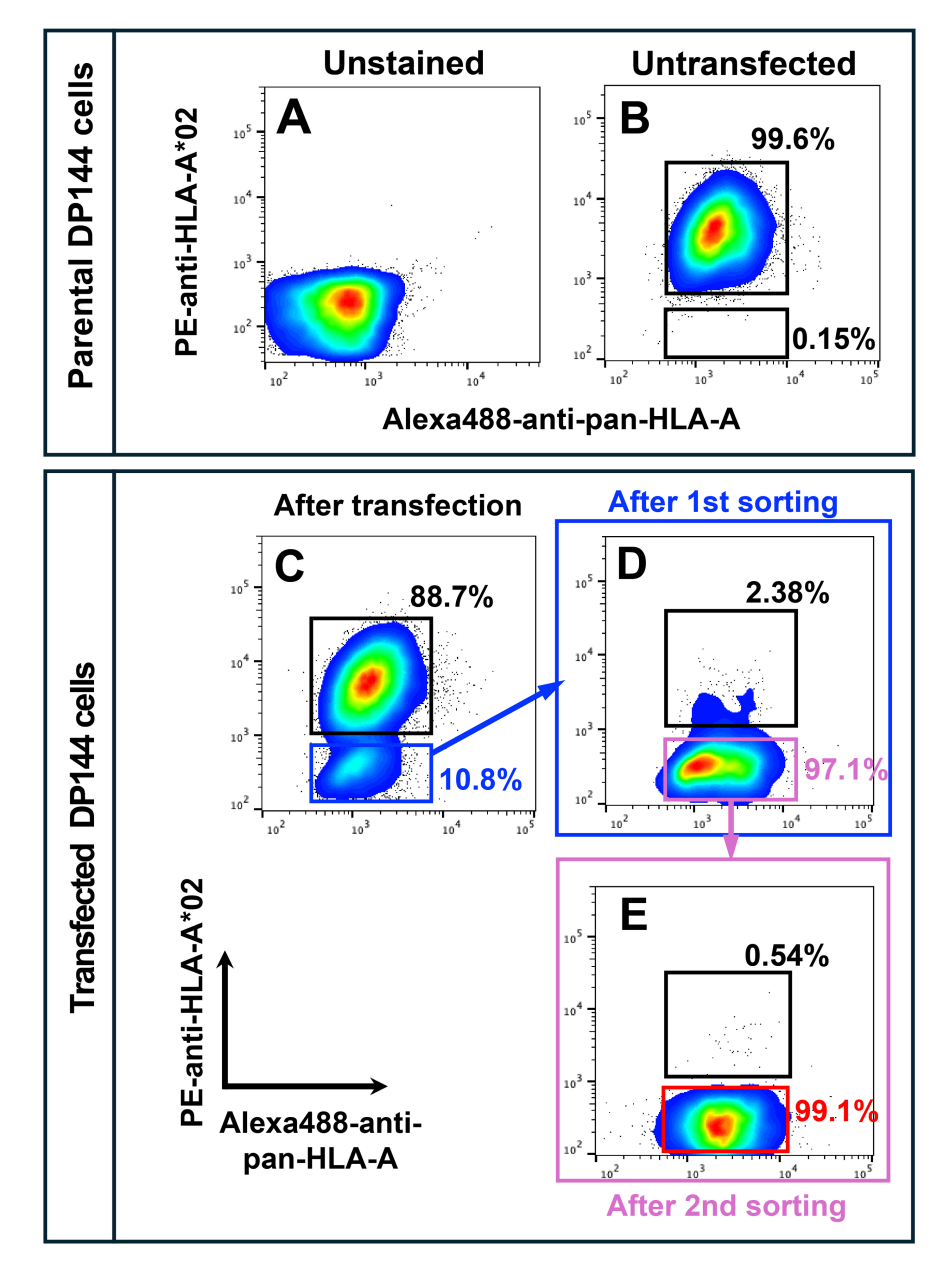
Flow cytometry-based enrichment of HLA-A*02-negative, HLA-A*33-positive cells A: Unstained DP144 cells (negative control) B: DP144 cells stained with anti-HLA-A*02 and anti-pan-HLA-A antibodies. The sorting gate for HLA-A*02-negative/pan-HLA-A-positive cells was determined based on this profile. C: Flow cytometric profile of DP144 cells 48 hours post-ZFN transfection. Approximately 10% of cells showed loss of HLA-A*02 expression while retaining pan-HLA-A expression (blue box). D: The FACS profile following the first round of cell sorting. Sorted cells were expanded and re-stained, showing ~97% enrichment of HLA-A*02-negative/pan-HLA-A-positive cells (pink box). E: Final FACS analysis after the second sorting cycle confirmed high-purity enrichment of the target population. HLA: Human leukocyte antigen, ZFN: Zinc finger nuclease, FACS: Fluorescence-activated cell sorting

To test the ability of the ZFNs to disrupt the HLA-A*02 protein expression in partially HHH DP144 cells, we transfected these cells with expression vectors encoding the HLAA-491a1/HLAA-r484a1 pair. Given the sequence similarity between the HLAA-491a1/HLAA-r484a1 target site and the corresponding region of the HLA-A*33:03 allele (Figure 1), we anticipated potential off-target effects on the HLA-A*33:03 allele. To specifically exclude cells with ablation of both HLA-A alleles, cells were double-stained with an anti-HLA-A*02 antibody and a pan-HLA-A monoclonal antibody, which recognizes a broad spectrum of HLA-A allomorphs, including the HLA-A*33 protein. Preliminary experiments confirmed the absence of interference between the antibodies in this double-staining protocol (Figures 2A-2B).

Flow cytometric analysis was performed to quantify the reduction in cell surface HLA-A*02 protein expression while maintaining HLA-A*33 expression. As intended, approximately 10% of the transfected cells lacked HLA-A*02 expression, confirming the efficacy of the ZFN pair in ablating HLA-A*02 expression (Figure 2C). 

To obtain highly purified pseudo-HHH DP144 cells, we performed two successive cycles of cell sorting to isolate the cells lacking HLA-A*02 expression while retaining HLA-A*33 expression. Therefore, cells negative for HLA-A*02 and positive for pan-HLA-A were sorted. Following two consecutive cycles of FACS-mediated cell purification, we successfully obtained the HLA-A*02-negative and pan-HLA-A-positive, i.e., HLA-A*33-positive cells with a purity exceeding 99% (Figures 2D-2E). These sorted cells are hereafter referred to as HLA-A*02-ablated cells. 

Molecular characterization of ZFN-induced modifications at the HLA-A locus

**Figure 3:**
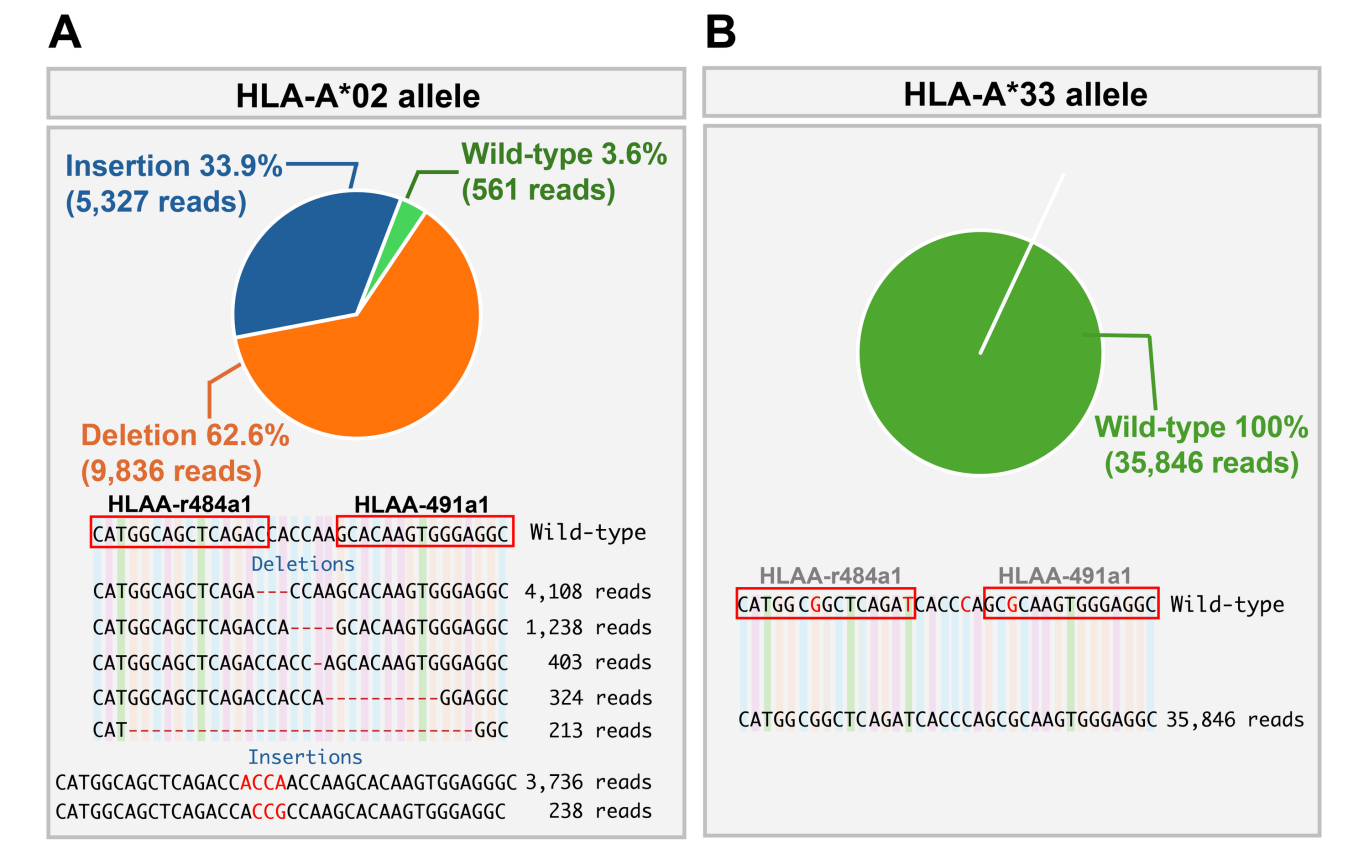
Targeted amplicon sequencing reveals allele-specific disruption of HLA-A*02 A: Indel profile at the HLA-A*02 locus in ZFN-edited cells. A pie chart indicates the proportion of insertion and deletion events. Read-depth plots confirm clustered indel events near the ZFN cleavage site. B: Analysis of the HLA-A*33 locus in the same cells revealed no detectable mutations, indicating high allele specificity of the ZFN pair. HLA: Human leukocyte antigen, ZFN: Zinc finger nuclease

To assess modifications at the target locus, allele-specific amplicon sequencing and Cas-Analyzer analysis [11] were performed on genomic DNA from HLA-A*02-ablated and wild-type DP144 cells. Deep sequencing of the ~200 bp region spanning the ZFN target site revealed indel mutations in over 96% of the HLA-A*02-ablated cell population (Figure 3A).

These indels, predominantly small insertions and deletions clustered near the predicted ZFN cleavage site (Figure 3A), are consistent with DNA double-strand break (DSB)-induced non-homologous end joining (NHEJ)-mediated repair. Notably, these indels were located in close proximity to the ZFN target site, providing strong evidence for ZFN-mediated disruption of the HLA-A*02 allele. In contrast, analysis of the corresponding region in the HLA-A*33 allele showed indel mutations below the detection limit (Figure 3B). 

Evaluation of potential off-target effects

**Figure 4:**
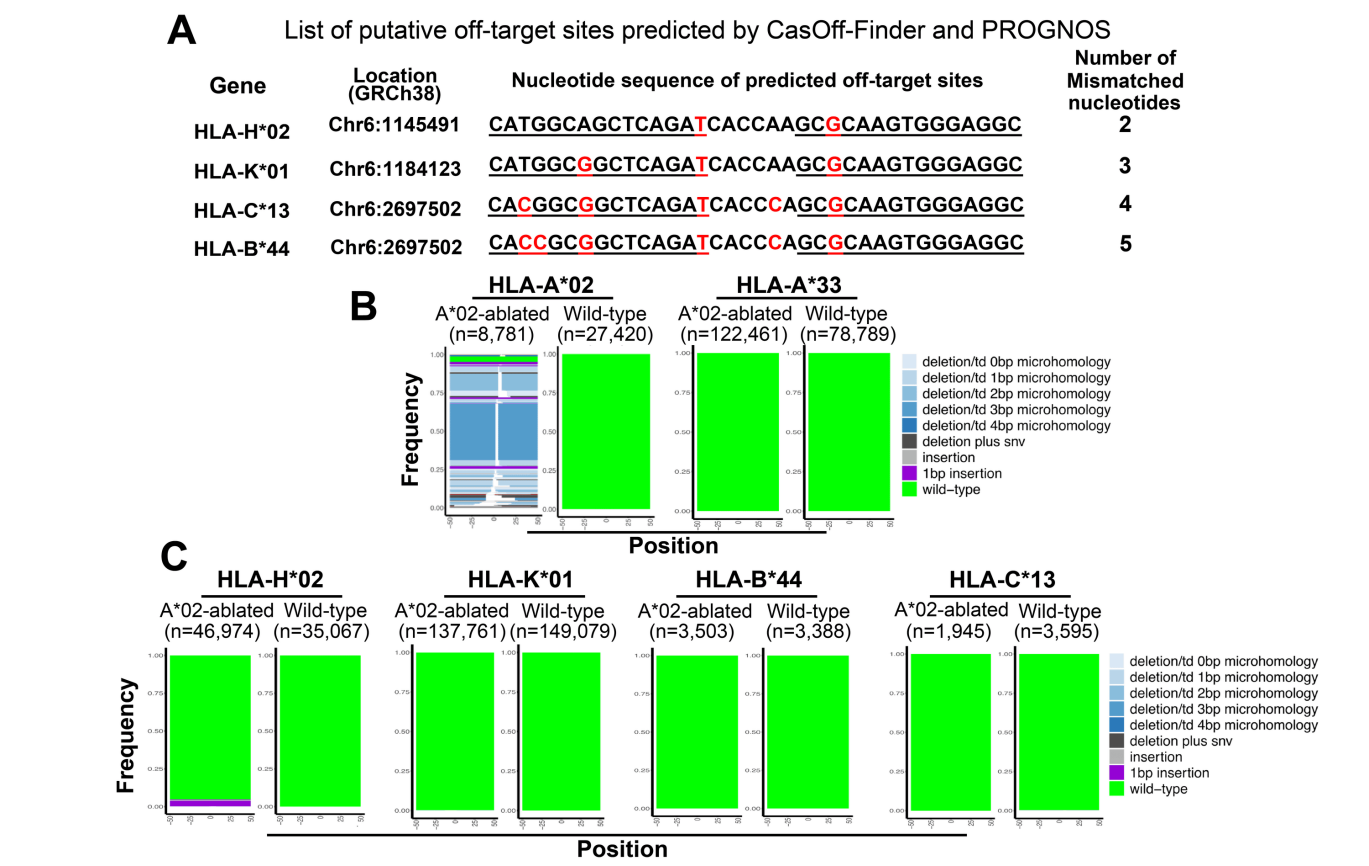
Evaluation of off-target effects at predicted ZFN binding sites A: List of predicted off-target sites identified using Cas-OFFinder and PROGNOS. Mismatched nucleotides are shown in red. B: The SIQ-generated tornado plots comparing mutational outcomes at HLA-A*02 and HLA-A*33 in ZFN-edited and wild-type cells. C: Tornado plots showing mutational profiles at other predicted off-target loci. Plot height reflects the proportion of each sequence variant among total reads. ZFN: Zinc finger nuclease, SIQ: Sequence interrogation and quantification, HLA: Human leukocyte antigen

To address the concern of potential off-target effects, we investigated the presence of indel mutations at predicted off-target genomic loci in HLA-A*02-ablated cells. Potential off-target sites were predicted using CasOff-Finder [22] and PROGNOS [23] analyses, allowing for up to five base mismatches and a 5-6 bp spacing distance between the HLAA-491a1 and HLAA-r484a1 recognition sequences. This analysis identified four potential off-target sites, including class I HLA genes and their putative pseudogenes (Figure 4A). 

The highest sequence similarity was observed between HLA-H*02 and the target site (two-base mismatch), while the lowest was between HLA-B*44 and the target site (five-base mismatch). The specific sequences of the predicted off-target sites are shown in Figure 4A. To determine whether indel mutations occurred at these potential off-target sites, we PCR-amplified ~200 bp genomic DNA fragments spanning each site. These fragments, along with positive (HLA-A*02) and negative (HLA-A*33) controls, were subjected to NGS-based targeted amplicon sequencing and analyzed using the SIQ software [12], which facilitates visualization of mutational profiles via SIQ PlotteR tornado plots [12]. Consistent with the Cas-Analyzer results, the SIQ analysis detected a range of indel mutations in the HLA-A*02 allele (Figure 4B) and confirmed the absence of mutations in the HLA-A*33 allele, validating the robustness of the SIQ assay. As anticipated from its high sequence similarity, the HLA-H*02 allele showed approximately 5% indel mutations, indicating a low-frequency off-target effect of the ZFN pair. By contrast, no significant indel formation was detected at the other predicted off-target sites (Figure 4C). These results indicate that off-target effects of the ZFN pair were minimal, limited primarily to the HLA-H*02 allele. 

Genome-wide assessment of genomic integrity

**Figure 5:**
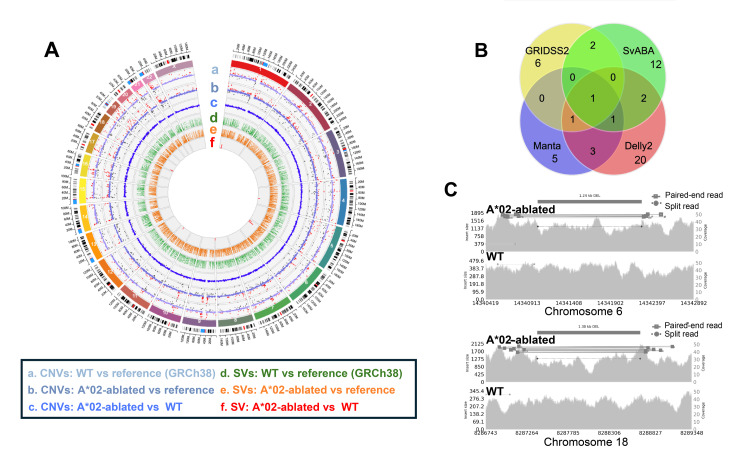
Genome-wide analysis of CNVs and SVs in HLA-A*02-ablated cells A: Circos plot summarizing CNV and SV distributions. Track a–c: Germline and somatic CNVs in wild-type and HLA-A*02-ablated cells relative to the GRCh38 reference. Red and black signals denote gains and losses (log2 scale: −1.0 to 2.0). Track d–f: Germline and somatic SVs shown as allele frequencies (range: 0–1.0). B: Venn diagram illustrating overlap among SVs identified by Manta, Delly, GRIDSS2, and SvABA. C: Samplot visualization of two high-confidence somatic deletions: a 1.24-kb intergenic deletion on chromosome 6 and a 1.30-kb intronic deletion on chromosome 18. Gray backgrounds represent read coverage. Solid and dotted bars denote supporting paired-end and split reads, respectively. CNVs: Copy number variations, SVs: Structural variants, HLA: Human leukocyte antigen

Given the growing concern regarding the potential risks associated with engineered nucleases, particularly their ability to induce DSBs that can result in large DNA deletions or chromosomal alterations [25], we sought to rigorously evaluate the genomic integrity of the HLA-A*02-ablated cells. To evaluate potential off-target effects and large-scale genomic alterations, PCR-free WGS was performed on HLA-A*02-ablated and control DP144 cells. Somatic CNV analysis using the CNVkit [15] did not reveal any obvious large-scale CNVs (>10 kbp) in the edited cells compared to controls (Figure 5A).

For detecting smaller structural variants (SVs > 50 bp), a consensus approach using four SV callers (Manta, Delly, GRIDSS2, SvABA) [16-19] was employed. This identified 10 potential SVs unique to the HLA-A*02-ablated cells (Figures 5A-5B; Table 2).

**Table 2:** Putative genomic structural variants in HLA-A*02-ablated cells identified by multiple SV callers SV: Structural variant, HLA: Human leukocyte antigen, *INV: Inversion, DEL: Deletion

Chromosome #	Start position	End position	SV type*	SV length (bp)	Allele frequency	SV called by
chr1	16037301	16060530	INV	23,229	0.03	Delly, GRIDSS2
chr1	21967428	21993051	DEL	25,601	0.141	Manta, Delly
chr4	124010588	124365223	INV	354,681	0.07	Manta Delly
chr6	14341037	14342273	DEL	1,236	0.11	Manta, Delly2, GRIDSS2, SvABA
chr6	14342272	14341038	DEL	1,236	0.016	GRIDSS2, SvABA
chr6	29793120	29927283	DEL	134,197	0.2	Manta, Delly, SvABA
chr8	143306981	143307972	INV	963	0.09	Delly, SvABA
chr11	98395998	98399225	DEL	3,179	0.074	Manta, Delly
chr18	8287394	8288697	DEL	1,302	0.12	Manta, GRIDSS2, SvABA
chr18	8288696	8287395	INV	1,302	0.18	GRIDSS2, SvABA

Manual curation via inspection of sequencing reads by Samplot [21] confirmed two high-confidence deletion-type SVs: a 1.24-kb deletion in an intergenic region on chromosome 6 and a 1.30-kb deletion within an intron of PTPRM on chromosome 18 (Figure 5C). These deletions did not overlap known coding regions, regulatory elements, nor any pathogenic variants listed in major genomic databases such as gnomAD [26], ClinVar [27], ClinGen [28], or dbVar [29]. Analysis of sequences flanking these deletion breakpoints (±100 bp) did not identify sequences homologous to the ZFN recognition site (data not shown). 

Validation of pseudo-HLA haplotype homozygosity by NGS HLA typing

**Figure 6:**
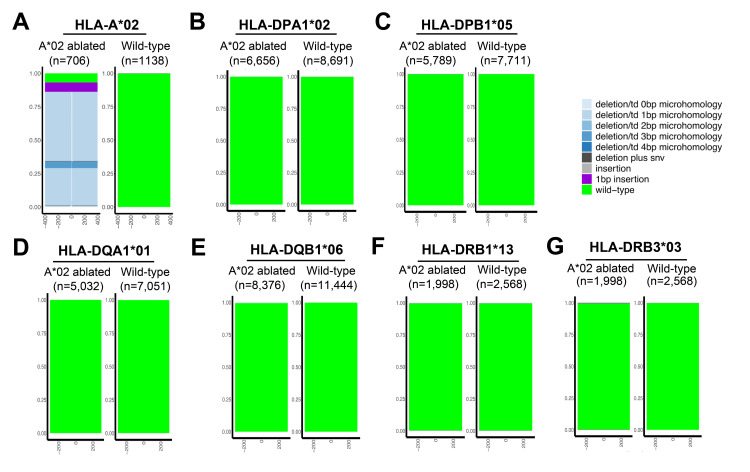
Genomic validation of HLA class II integrity in HLA-A*02-ablated cells using long-range NGS A: Tornado plots show indel mutations at the HLA-A*02 locus in ZFN-edited cells, while no mutations are observed in wild-type controls. B-G: Tornado plots confirm the absence of mutations at the following class II HLA loci: DPA1*02, DPB1*05, DQA1*01, DQB1*06, DRB1*13, and DRB3*03, demonstrating preserved sequence integrity after editing. Plot height corresponds to read abundance for each variant (n). HLA: Human leukocyte antigen, NGS: Next-generation sequencing, ZFN: Zinc finger nuclease

As final validation, we sought to genetically verify their pseudo-HHH status. To examine the genetic status of the HLA loci in the edited cells, long-range PCR (>5 kbp) coupled with NGS-based HLA typing was performed for 11 clinically relevant HLA loci. Sequence analysis using the SIQ tool [12] confirmed the presence of numerous mutations across the coding region of the HLA-A*02 allele in the ablated cells, consistent with the amplicon sequencing results, whereas no such mutations were found in wild-type controls (Figure 6A), verifying the effectiveness of this approach.

Importantly, sequence analysis of the HLA-A*33 allele and the other assessed class I (HLA-B*44, HLA-C*13) and class II alleles (HLA-DPA1*02, -DPB1*05, -DQA1*01, -DQB1*06, -DRB1*13, -DRB3*03) showed no detectable mutations within their coding regions in the HLA-A*02-ablated cells (Figure 4B, Figure 6B-6G). These findings validate that the HLA-A*02-ablated DP144 cells possess genetically confirmed pseudo-HLA haplotype homozygosity. However, it remains to be determined whether these cells meet the criteria for a functional HHH status. 

## Discussion

This study successfully demonstrates the feasibility of generating pseudo-HHH human DPCs through allele-specific gene editing using ZFNs. We achieved efficient and highly specific disruption of the targeted HLA-A*02:01 allele in DP144 cells, which were heterozygous at HLA-A but homozygous at other key loci, while preserving the non-targeted HLA-A*33:03 allele and other essential HLA class I and II genes. These findings directly address the challenge outlined in the introduction regarding the limited availability of HHH donors for DPC-based therapies by providing a proof-of-concept for artificially creating HLA-compatible cells. 

Our study builds upon pioneering efforts to enhance immune compatibility in allogeneic therapies through HLA gene editing. Torikai et al. demonstrated that eliminating HLA-A expression in hematopoietic stem cells using ZFNs could broaden donor compatibility, particularly benefiting patients from underrepresented populations [30]. However, complete ablation of HLA-A may modulate immune responses, including natural killer (NK) cell-mediated cytotoxicity. To address this, Xu et al. employed CRISPR/Cas9 to selectively delete HLA-A and -B while retaining HLA-C, producing pseudo-homozygous induced pluripotent stem cells (iPSCs) capable of evading both CD8+ T cell and NK cell recognition [8]. Despite this success, the reliance on protospacer adjacent motif (PAM) sequences restricts the flexibility of CRISPR/Cas9 in targeting highly polymorphic HLA alleles. 

In contrast, our study used ZFNs to achieve precise, allele-specific disruption of HLA-A*02:01 in primary DPCs. The absence of PAM constraints allows ZFNs to target individual HLA alleles more flexibly. Importantly, the use of primary DPCs, rather than iPSCs, avoids tumorigenicity concerns and supports safer clinical translation. Given a broad differentiation capability of DPCs, our strategy offers a complementary and potentially more practical approach to creating immune-compatible cells for regenerative medicine. The choice of ZFNs over CRISPR/Cas9 was strategic, leveraging their flexibility in targeting sequences without strict PAM requirements and their ability to be designed for high specificity, which is crucial when distinguishing between highly homologous HLA alleles. Our results validate this approach, showing effective knockout of HLA-A*02 with negligible impact on the HLA-A*33 allele at the target site, as confirmed by deep sequencing. This level of specificity is paramount for generating functional pseudo-HHH cells where only one specific allele needs modification. 

A critical aspect of evaluating any gene-editing strategy for clinical translation is the assessment of genomic integrity and off-target effects. Our comprehensive WGS analysis, employing PCR-free library preparation and a multi-caller consensus approach for SV detection, aimed to provide a rigorous evaluation. While no large-scale CNVs were detected, the analysis did identify two high-confidence, small deletion-type SVs in intergenic and intronic regions. Importantly, these SVs did not overlap coding or known regulatory regions, and no ZFN recognition sequences were found near the breakpoints, making their direct attribution to off-target ZFN activity uncertain; they could potentially represent spontaneous mutations arising during cell culture. Nevertheless, their presence underscores the absolute necessity for meticulous, genome-wide surveillance of edited cells. While our targeted sequencing of predicted off-target sites showed no indels other than HLA-H allele, WGS has limitations in detecting low-frequency point mutations or small indels outside these predicted areas. Therefore, future safety assessments should incorporate higher sensitivity, unbiased methods like genome-wide unbiased identification of DSBs enabled by sequencing (GUIDE-Seq) [31] or digenome-seq [32] to provide a more complete picture of potential off-target events before clinical application. 

Despite the promising results regarding specificity at the target site, the identification of SVs and the inherent risks associated with DSB-dependent editing tools (e.g., ZFNs, transcription activator-like effector nucleases (TALENs), CRISPR/Cas9), such as potential large deletions or chromosomal rearrangements, highlight the need for exploring alternative strategies. Newer non-cleavage-based technologies like base editing [33] and prime editing [34], which allow precise nucleotide changes without inducing DSBs, represent potentially safer avenues for future development of pseudo-HHH DPCs. Refining these techniques could mitigate risks associated with genomic instability, enhancing the prospects for regulatory approval and clinical use.

While this study establishes the principle of ZFN-mediated pseudo-HHH DPC generation, limitations remain. The scalability of producing multiple pseudo-HHH lines to cover a diverse population requires further investigation, including identifying the most impactful HLA alleles to target for maximal population coverage. Furthermore, the long-term stability and functional equivalence of these edited cells compared to unmodified DPCs need thorough characterization. Cost-effectiveness will also be crucial for widespread clinical adoption, necessitating streamlined and efficient production protocols. 

In conclusion, this work provides a significant step towards overcoming HLA barriers in DPC transplantation by demonstrating successful ZFN-mediated generation of pseudo-HHH cells. The ZFNs offer advantages for targeting complex, polymorphic regions like HLA compared to CRISPR/Cas9. However, careful consideration of off-target effects and genomic stability is essential. Future efforts should focus on employing high-sensitivity off-target detection methods and exploring non-DSB editing technologies, alongside addressing scalability and cost, to advance the development of safe and effective "off-the-shelf" DPC therapies for regenerative medicine. 

Study limitations and future directions

This study demonstrates that pseudo-HHH cells can be generated using ZFNs, but the presence of off-target mutations, particularly genomic SVs, remains a significant concern. The complexity of the HLA region, which contains numerous homologous sequences and pseudogenes, makes it prone to unintended genomic alterations when using DSB-based editing tools. Indeed, the implication of genomic alterations within HLA loci has been demonstrated in various autoimmune and infectious diseases [35]. Additionally, the inherent genomic instability of DPCs may have contributed to some of the observed structural variations. Future research should explore non-cleavage-based editing strategies to minimize these risks while maintaining efficient gene modification.

Another limitation is the scalability of the approach. While genome editing allows for the artificial expansion of HLA-compatible donor cells, it remains unclear how many pseudo-HHH cell lines would be needed to provide sufficient HLA coverage across a diverse patient population. Future studies should focus on identifying the most relevant HLA alleles for modification and developing population-specific donor cell banks to maximize compatibility.

A final challenge is the cost-effectiveness of genome-edited donor cell production. Large-scale implementation will require streamlined, cost-efficient protocols to ensure accessibility for clinical applications. Advances in high-throughput genome editing and cell culture technologies will be essential to making this approach feasible for widespread use in regenerative medicine.

## Conclusions

This proof-of-concept study demonstrates the feasibility of generating pseudo-HHH DPCs using ZFNs as an alternative to CRISPR/Cas9, providing a flexible and targeted approach for modifying the HLA loci. While CRISPR/Cas9 has been successfully applied in iPSCs for similar purposes, its dependence on a PAM sequence limits its versatility in targeting polymorphic genomic regions like HLA. The ZFNs offer greater flexibility in target site selection, making them a promising alternative gene-editing tool for precise HLA modification.

However, our findings also highlight key technical and practical challenges that must be addressed before genome-edited donor cells can be widely implemented. The risk of off-target effects, particularly large deletions and structural variations, must be mitigated. Additionally, the limited availability of partial HHH donors remains a significant barrier, requiring carefully designed gene-editing strategies tailored to population-specific HLA distributions. By advancing gene-editing methodologies and expanding the donor pool, this approach has the potential to revolutionize allogeneic cell transplantation, paving the way for the next generation of immune-compatible, off-the-shelf cell therapies.
